# Using a mobile application for antiretroviral therapy adherence in people living with HIV: A longitudinal pilot study

**DOI:** 10.4102/sajhivmed.v26i1.1646

**Published:** 2025-01-31

**Authors:** Rejane Caetani, Susana L. Wiechmann, Jacques D. Brancher, Vitor H.F. Oliveira, Rafael Deminice

**Affiliations:** 1 Health Sciences Center, State University of Londrina, Londrina, Brazil; 2 Department of Clinical Medicine, State University of Londrina, Londrina, Brazil; 3 Department of Computer Science, State University of Londrina, Londrina, Brazil; 4 Department of Biobehavioral Nursing and Health Informatics, University of Washington, Seattle, United States of America; 5 Department of Physical Education, State University of Londrina, Londrina, Brazil

**Keywords:** HIV, antiretroviral therapy, adherence, mobile applications, mHealth

## Abstract

**Background:**

The success of HIV treatment hinges on consistent adherence to antiretroviral therapy (ART).

**Objectives:**

To conduct a longitudinal pilot study to assess the feasibility, acceptability and effectiveness of a mobile app to improve ART adherence.

**Method:**

This study included adults living with HIV and using ART, who were allocated into two groups according to their willingness to use the app: users of the mobile application for ART management (Mobile) and non-users (Control). The application was developed by the researchers, and uses an alarm system to record ART use. Adherence was also assessed using the ‘*Cuestionario para la Evaluación de la Adhesión al Tratamiento Antiretroviral*’ (CEAT-VIH) and the Multi-Method Tool questionnaire. Another questionnaire was administered to application users to assess acceptability. After 90 days, all the questionnaires were reapplied.

**Results:**

A significant difference in adherence was observed between the Mobile and Control groups (*P* = 0.04), but there was no significant difference in time (*P* = 0.2) or interaction (*P* = 0.5).

**Conclusion:**

The application was not effective in improving ART adherence and showed low viability, but was considered acceptable among the participants.

**What this study adds:** This study describes a pilot test carried out with an application for ART adherence in people living with HIV, which provides automated feedback to researchers.

## Introduction

Antiretroviral therapy (ART) has transformed the trajectory of HIV infection, once considered fatal, into a manageable chronic condition. As ART continues to advance, the life expectancy of people living with HIV (PLWH) approaches that of individuals without HIV.^1,2^ Effective ART usage results in the suppression of viral load (VL) in the bloodstream, rendering HIV undetectable and, consequently, non-transmissible sexually.^3^ However, achieving an undetectable VL requires PLWH to maintain adherence to ART.^4^ Lack of adherence to ART can lead to treatment failure and the development of drug-resistant strains of HIV, worsening health outcomes, including increased risk of opportunistic infections and progression to AIDS, increased risk of virus transmission, negative impacts on overall quality of life, and increased healthcare costs associated with HIV infection complications.^4,5,6^ Therefore, strategies for improving and maintaining adherence to ART are crucial.

Technological advances have led to the concept of mobile health (mHealth), which involves using internet-based mobile applications (apps) to support medical and health activities.^7^ As of 2017, more than 50% of smartphone users worldwide had installed mobile health apps.^8^ Generally, these apps help to manage diet and medication intake and physical activity practices, provide medical references, and assist in medical decision-making. Regarding PLWH, previous investigations have shown that the use of apps, short message services, and phone calls was considered acceptable to improve ART adherence in American,^9,10^ European,^11^ African,^12^ and Asian countries.^13^

The 2021 Continuous National Household Sample Survey in Brazil showed that internet usage reached 90% of households nationwide, with mobile phones being the primary devices for accessing the internet at home.^14^ Despite its high potential, the use of smartphones and health application devices has encountered certain limitations in Brazil. While messaging apps such as WhatsApp are widely popular, they lack automatic feedback mechanisms, and rely on health agents to send and monitor messages. Additionally, most commercially available ART intake reminders and apps are produced outside of Brazil and do not take Brazilian regulations into account.

Therefore, in 2020, our research group developed a mobile app for ART self-management, called Uelness. This app was designed in Brazilian Portuguese, considering Brazilian regulations and particularities, and automatically provides feedback on ART adherence. Therefore, this study aimed to conduct a pilot study to assess the feasibility, acceptability, and effectiveness of the Uelness app in improving ART adherence.

## Research methods and design

### Study design and procedures

We conducted a longitudinal pilot study to test the feasibility, acceptability, and effectiveness of a mobile app for ART adherence. In collaboration with the hospital staff, researchers approached potential participants after their routine clinical care visit and invited them to participate in the study. Upon acceptance, the patients received an explanation of the study procedures and signed an informed consent form. Subsequently, all participants underwent face-to-face interviews, during which they were asked about their socioeconomic status, demographic information, and ART adherence.

Participants were briefed on the Uelness app, and those who consented had it installed on their mobile phones. All participants received assistance installing, registering, and using the app. During the subsequent week, the researchers contacted all participants and established a support channel for using the app. At least two contacts were made with each participant. After 90 days, the researchers telephoned all the participants to reassess their ART adherence using questionnaires. At least three attempts were made to reach each participant. Additionally, Uelness users completed a questionnaire to evaluate the app, focusing on its acceptability.

Participants’ medical records were used to collect information on the year of HIV diagnosis, composition and date of ART initiation, presence of comorbidities and opportunistic infections, use of tobacco, alcohol and illicit drugs, blood pressure measurements, and biochemical test results (HIV VL, CD4+ T-lymphocytes, fasting glucose, glycated haemoglobin, serum creatinine, alanine aminotransferase, aspartate aminotransferase, haemoglobin, and lipid profile). We considered the blood test results carried out closest to the study date. The blood test results selected for the pre-study period were collected approximately 1 week before the study evaluations and, for the post-study period, we considered the blood test results carried out closest to the end of the study, given that patients followed up at the outpatient clinic usually have their tests repeated every 6 months.

### Participants

Between March and December 2022, participants were approached at the HIV Outpatient Clinic of the University Hospital of the State University. The inclusion criteria for the study were: (1) having a diagnosis of HIV infection noted in their medical records; (2) being on ART; (3) not having a physical or intellectual disability that made it impossible to answer the collection instruments; (4) being over 18 years of age; (5) having a mobile phone compatible with the app, with an Android operating system.

The participants were then allocated into two groups by convenience sampling, according to their willingness to use the mobile app: ART management mobile app users (Mobile) and non-users (Control). Both groups continued with their standard treatment provided by the HIV Outpatient Clinic at the University Hospital.

### Mobile application

The Uelness app was developed by researchers from the university itself. The app’s primary goal is to allow PLWH to self-manage ART, with functions such as reminders to take their daily medication, notifying when ART is running low for a new refill, scheduling medical appointments, and automatically collecting, storing, and transferring ART consumption information to the researchers.

In addition, the app also provides information on Body Mass Index (BMI) and a daily step and calorie counter to improve interest and adherence to the app. The app was provided for free for the Android operating system, and a website link was also provided (https://movimentoexcan.wixsite.com/uelness). The app has been registered with the National Institute of Industrial Production, with the following patent: “Uelness. 2022. Patent: Computer Program”.

### Determining antiretroviral therapy adherence

Two instruments were used to assess participants’ adherence to ART: The Multi-Method Tool^15^ and the ‘*Cuestionario para la Evaluación de la Adhesión al Tratamiento Antiretroviral*’ (CEAT-VIH), a multidimensional questionnaire which has been adapted for Brazilian Portuguese.^16,17^ The mobile app also measured the Mobile group’s adherence to ART.

The Multi-Method Tool comprises a combination of three measurements: self-reporting of ART usage, a visual analogue scale, and a pill identification test. Overall adherence was assessed using a composite adherence score, which is calculated as the sum of the scores from each of the three measures, and categorises adherence as high, moderate, or low.^15^

The CEAT-VIH questionnaire consists of questions in which PLWH self-evaluate their adherence to medication, history of non-adherence, doctor-patient communication, beliefs about ART, beliefs and expectations about therapeutic efficacy, efforts to follow the treatment, evaluation of side effects, and level of satisfaction. Each question was assigned an individual score, and the total score was obtained by their sum (ranging from a minimum of 17 and a maximum of 89). Participants were classified as having either low/insufficient adherence (raw score ≤ 74, percentile ≤ 49%), good/adequate adherence (raw score 75 – 79; percentile 50% – 85%), or strict adherence (raw score ≥ 80, percentile ≥ 85%).^16,17^

PLWH in the Mobile group also had their ART adherence measured by Uelness. The app tracked ART intake through an alarm and response system that sent reminders when it was time to take the medication. The user then indicated whether they had taken their medication by clicking the ‘YES’ or ‘NO’ button. A postponement button was also included, allowing a reminder to be postponed every 20 min until the user chose either ‘YES’ or ‘NO’. If the user did not respond to any option, the app logged a ‘no medication’ event. Based on these responses, the app generated a report for the researchers indicating the percentage of positive responses, used to assess ART adherence. The monitoring period for users using the app lasted for 90 days.

### Acceptability

Participants who used Uelness completed an evaluation questionnaire focusing on acceptability. For the open-ended questions, we carried out an inductive thematic analysis^18^ to identify obstacles and facilitators. Participants’ responses were compiled in a separate spreadsheet and categorised into themes to aid interpretation.

### Outcomes

The primary outcome was the effectiveness of Uelness in increasing ART adherence and its association with clinical and general health parameters.

The secondary outcomes included assessing the feasibility and acceptability of the app. Feasibility was measured through engagement using the number of apps installed, the number of users, and the time of use. Acceptability was evaluated based on the user’s evaluation of the app.

### Statistical analysis

Descriptive statistics were presented using mean, median, and interquartile ranges for continuous variables, and absolute numbers and percentages for categorical variables. The Shapiro-Wilk test was used to test the normality of the data. Student’s *t*-test and the Mann-Whitney U test were used to check for possible differences between the groups, for parametric and non-parametric variables, respectively. Fisher’s exact and the chi-squared tests were used to evaluate the association between the groups for the categorical variables. A two-way analysis of variance (ANOVA) was performed to compare ART adherence between the Mobile and Control groups before and during the study period. The two-way ANOVA test for parametric data and the McNemar test for categorical variables were used to compare clinical and health parameters between the Control group and the group (Mobile) that used the Uelness app, before and after 90 days of follow-up. In all cases, significance was set at *P* < 0.05, and all statistical tests were carried out using GraphPad Prism version 8.0.1 (GraphPad Software, Boston, Massachusetts, United States). For the open-ended questions regarding app acceptability, we conducted an inductive thematic analysis to identify barriers and facilitators. Participants’ responses were compiled in a separate spreadsheet and categorised into themes to aid interpretation.

### Ethical considerations

Ethical clearance to conduct this study was obtained from the State University of Londrina Ethics Committee for Research Involving Human Beings (reference no.: CAAE: 38167920.7.0000.5231). Written informed consent was obtained from all participants prior to all study procedures.

## Results

### Participants

Four hundred and nine (409) individuals were approached at the outpatient clinic. Of these, 100 agreed to participate, and 95 met the study inclusion criteria. These were divided into users of mobile ART management apps (Mobile, *n* = 52) and non-users (Control, *n* = 43).

**FIGURE 1:**
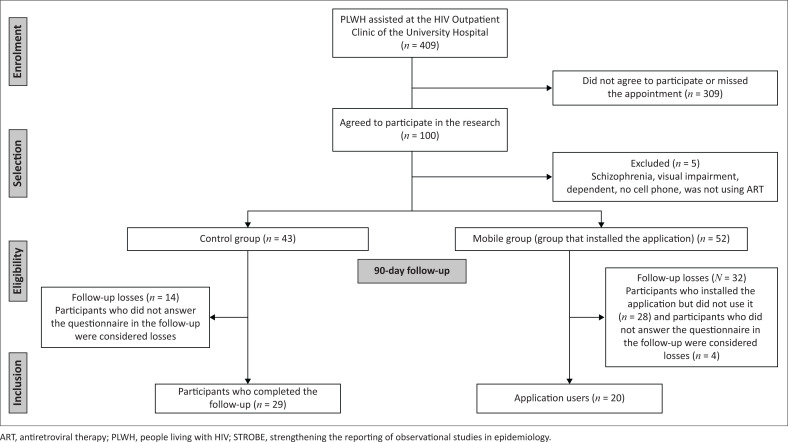
STROBE flowchart for recruitment and selection of participants.

After 90 days of follow-up, 20 participants (38%) in the Mobile group had used the app at least once during the study period and had completed the subsequent surveys and adherence evaluation. In contrast, 14 participants were excluded from the Control group for not completing the 90 days of post-surveys and adherence evaluation, leaving 29 participants in the Control group. Figure 1 shows the Strengthening the Reporting of Observational Studies in Epidemiology (STROBE) flowchart illustrating the recruiting and selection of participants.

The characteristics of the participants are shown in Table 1. The participants included in the analysis were 49 ± 13 years old, time since HIV diagnosis was 13 (6 – 25) years and they had been using ART for a similar length of time, most had an undetectable VL (< 40 copies/mL; *n* = 44% – 90%) and CD4+ T-lymphocytes > 350 cells/mm^3^, and were taking nucleoside reverse transcriptase inhibitor + integrase chain transferase inhibitor (NRTI + INSTI; *n* = 16, 33%) and nucleoside reverse transcriptase inhibitor + protease inhibitor (NRTI + PI; *n* = 16, 33%). The majority of participants were women, white, and without a steady partner. No differences were found between the Mobile and the Control groups in terms of age, sociodemographic variables, immunological and virological parameters, composition of ART regimen, and the most prevalent comorbidities.

**TABLE 1:** General characteristics of people living with HIV, divided into a Control group and a group that used the Uelness application (Mobile).

Variables	Total (*N* = 49)				Control (*n* = 29)				Mobile (*n* = 20)				*P*
	*n*	%	Median	IQR	*n*	%	Median	IQR	*n*	%	Median	IQR	
**Gender**	-	-	-	-	-	-	-	-	-	-	-	-	0.29
Men	19	39	-	-	13	45	-	-	6	30	-	-	-
Women	30	61	-	-	16	55	-	-	14	70	-	-	-
**Ethnicity**	-	-	-	-	-	-	-	-	-	-	-	-	0.75
White	24	49	-	-	13	44	-	-	11	55	-	-	-
Black	13	27	-	-	8	28	-	-	5	25	-	-	-
Other†	12	24	-	-	8	28	-	-	4	20	-	-	-
**Marital status**	-	-	-	-	-	-	-	-	-	-	-	-	0.31
Single, widowed, divorced	37	76	-	-	20	69	-	-	17	85	-	-	-
Married, stable union	12	24	-	-	9	31	-	-	3	15	-	-	-
**Level of education (years)**	-	-	-	-	-	-	-	-	-	-	-	-	0.39
< 8	21	43	-	-	14	48	-	-	7	35	-	-	-
≥ 8	28	57	-	-	15	52	-	-	13	65	-	-	-
**Monthly income**	-	-	-	-	-	-	-	-	-	-	-	-	0.37
< 1 minimum wage	19	39	-	-	13	45	-	-	6	30	-	-	-
≥ 1 minimum wage	30	61	-	-	16	55	-	-	14	70	-	-	-
**Clinical (years)**	-	-	-	-	-	-	-	-	-	-	-	-	-
Time since HIV diagnosis	-	-	13	6–25	-	-	10	6–21	-	-	18	7–26	0.15
Time of ART use	-	-	13	6–23	-	-	10	6–17	-	-	18	6–25	0.16
**HIV viral load (copies/mL)**	-	-	-	-	-	-	-	-	-	-	-	-	0.63
Undetectable (< 40)	44	90	-	-	25	86	-	-	19	95	-	-	-
Detectable (> 40)	5	10	-	-	4	14	-	-	1	5	-	-	-
**CD4^+^ T-lymphocyte count (cells/mm^3^)**	-	-	-	-	-	-	-	-	-	-	-	-	-
CD4^+^ T-lymphocytes	-	-	573	372–850	-	-	460	277–756	-	-	626	504–896	0.14
CD4^+^ T-lymphocyte nadir	-	-	96	35–256	-	-	52	30–220	-	-	180	47–283	0.15
**ART regimen**	-	-	-	-	-	-	-	-	-	-	-	-	0.54
NRTI+INSTI	16	33	-	-	9	31	-	-	7	35	-	-	-
NRTI+PI	16	33	-	-	8	28	-	-	8	40	-	-	-
NRTI+NNRTI	11	22	-	-	7	24	-	-	4	20	-	-	-
Other‡	6	12	-	-	5	17	-	-	1	5	-	-	-

Note: Age in years (mean ± s.d.): General, 49 ± 13; Control, 50 ± 13; Mobile, 47 ± 14. Age in years, *P* = 0.47. Interquartile range reflects the range between the 25th and 75th percentile.

IQR, interquartile range; ART, antiretroviral therapy; FI, fusion inhibitor; INSTI, integrase chain transferase inhibitor; NRTI, nucleoside reverse transcriptase inhibitor; INSTI, integrase chain transferase inhibitor; PI, protease inhibitor; NNRTI, non-nucleoside reverse transcriptase inhibitor; s.d.; standard deviation.

† , Other includes brown, indigenous and Asian people;

‡ , Other includes: NRTI+PI+INSTI, NRTI+PI+FI, and NRTI+NNRTI+PI.

### Antiretroviral therapy adherence and clinical and general health parameters

**FIGURE 2:**
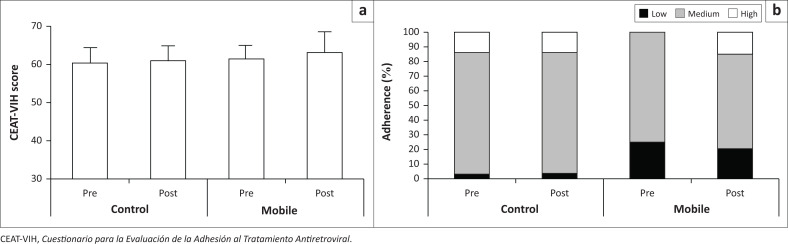
Adherence to antiretroviral therapy (ART) assessed using the ‘*Cuestionario para la Evaluación de la Adhesión al Tratamiento Antiretroviral*’ (CEAT-VIH) (a) and the Multi-Method Tool (b) for the Control and Mobile groups, pre and post 90-day follow-up using or not using the Uelness application for ART self-management. *P* < 0.05 compared to pre, using two-way analysis of variance. The values in (a) are expressed as mean and standard deviation and in (b) as percentage (%).

The results of ART adherence, determined by the Multi-Method Tool and the CEAT-VIH questionnaire for both the Mobile and Control groups, are presented in Figure 2. According to the Multi-Method Tool, 75% of participants in the Mobile group and 83% in the Control group were classified as having medium adherence to ART, with no changes in ART adherence after 90 days of using or not using Uelness (*P* = 0.2 and *P* = 0.9, respectively) (Figure 2b). Regarding the CEAT-HIV questionnaire, the participants were classified as having insufficient adherence. A significant difference in adherence was observed between the Mobile and Control groups (*P* = 0.04), but there was no significant difference in time (*P* = 0.2) or interaction (*P* = 0.5), as shown in Figure 2a.

The effects of using Uelness on certain clinical and general health parameters are shown in Table 2. At the start of the study, 95% of the participants in the Mobile group and 90% in the Control group had an undetectable HIV VL. After the 90-day study period, all participants in the Mobile group had an undetectable VL, while in the Control group, one individual who had an undetectable VL became detectable and discontinued conventional treatment at the HIV Outpatient Clinic.

**TABLE 2:** Comparison of clinical and health parameters between the Control group and the group that used the Uelness application (Mobile) pre and post 90-day follow-up.

Medical record extraction	Control (*n* = 29)									Mobile (*n* = 20)									*P*-value‡		
	Pre				Post				%change	Pre				Post				%change	Time	Group	Interaction
	*n*	%	Median	IQR	*n*	%	Median	IQR		*n*	%	Median	IQR	*n*	%	Median	IQR				
**HIV viral load (copies/mL) †**	-	-	-	-		-	-	-	-	-	-	-	-	-	-	-	-	-	-	-	-
Undetectable (< 40)	26	90	-	-	25	86	-	-	−4	19	95	-	-	20	100	-	-	5	-	-	-
Detectable (> 40)	3	10	-	-	4	14	-	-	4	1	5	-	-	0	0	-	-	−100	-	-	-
**CD4^+^ T-lymphocyte count (cells/mm^3^)** †	-	-	460.0	298–756	-	-	418.0	277–756	−10	-	-	626.0	504–896	-	-	626.0	504–896	0	0.50	0.01	0.02
0–200	2	7	-	-	0	0	-	-	-	0	0	-	-	0	0	-	-	-	-	-	-
200–350	7	24	-	-	10	34	-	-	-	1	5	-	-	1	5	-	-	-	-	-	-
> 350	20	69	-	-	19	66	-	-	-	19	95	-	-	19	95	-	-	-	-	-	-
Fasting glucose (mg/dL)	-	-	95.0	88–99	-	-	93.0	88–101	−2	-	-	92.0	87–97	-	-	93.0	86–98	1	0.97	0.68	0.53
HbA1c (%)	-	-	5.4	5.0–5.8	-	-	5.4	5.1–6.1	0	-	-	5.4	4.9–5.7	-	-	5.3	5-5.5	−2	0.65	0.21	0.39
Serum creatinine (mg/dL)	-	-	0.9	0.9–1.1	-	-	1.0	0.8–1.1	10	-	-	0.8	0.7–1.0	-	-	0.9	0.7–1.1	12	0.72	0.01	0.72
Total cholesterol (mg/dL)	-	-	200.0	169–228	-	-	200.0	169–215	0	-	-	199.0	167–228	-	-	199.0	162–212	2	0.66	0.92	0.64
HDL-c cholesterol (mg/dL)	-	-	49.0	43–62	-	-	52.0	40–66	6	-	-	45.0	40–59	-	-	50.0	43–60	11	0.23	0.32	0.70
LDL-c cholesterol (mg/dL)	-	-	111.0	91–131	-	-	111.0	85–127	0	-	-	118.0	77–174	-	-	114.0	85–135	−3	0.27	0.32	0.67
Triglycerides (mg/dL)	-	-	96.0	76–182	-	-	93.0	69–169	−3	-	-	119.0	97–176	-	-	125.0	81–169	−5	0.43	0.53	0.86
Haemoglobin (g/dL)	-	-	14.3	13–15.5	-	-	14.6	13.1–15.5	2	-	-	14.0	12.7–15.3	-	-	14.1	13.4–15.1	0	0.77	0.57	0.77
AST (U/L)	-	-	20.0	16–29	-	-	22.0	19–27	10	-	-	24.0	19–26	-	-	24.0	20–33	0	0.16	0.19	0.21
ALT (U/L)	-	-	29.0	22–44	-	-	28.0	23–34	3	-	-	26.0	22–36	-	-	28.0	25–33	8	0.98	0.19	0.93
Systolic arterial pressure (mmHg)	-	-	120.0	110–130	-	-	120.0	110–138	0	-	-	128.0	119–146	-	-	122.0	113–138	−5	0.70	0.02	0.28
Diastolic arterial pressure (mmHg)	-	-	70.0	60–80	-	-	70.0	65–80	0	-	-	80.0	67–86	-	-	67.0	60–79	−19	0.41	0.13	0.05

Note: Interquartile ranges were expressed as 25th – 75th percentile.

ALT, alanine aminotransferase; AST, Aspartate Aminotransferase; HDL-c, high-density lipoprotein; IQR, interquartile range; LDL-c, low-density lipoprotein; HbA1c; Glycosylated haemoglobin; VL, viral load.

† , *P* = 0.03, *P*-value calculated using Mc Nemar test;

‡ , *P*-value calculated using two-way analysis of variance.

Over time, there was a reduction in the CD4+ T-lymphocyte count in the Control group, while this was maintained in the Mobile group, with *P* = 0.01 and interaction *P* = 0.02. There was a decrease in systolic blood pressure in the Mobile group (*P* = 0.02), which was not observed in the Control group. Regarding diastolic blood pressure, there was an interaction, with *P* = 0.05. No significant differences were found between the Mobile and Control groups for the other parameters assessed.

### Viability and acceptance of the application

Out of the 95 study participants, 52 (55%) initially agreed to install the app on their mobile phones. Among these, 32 (61%) never registered any ART usage and were classified as non-users. The remaining 20 participants recorded their ART use for the first 30 days, achieving an average ART adherence rate of 100%. After 60 days of follow-up, only 13 participants (14%) were actively recording their ART usage, with an average adherence rate of 64%. By the end of the 90 days, the number of participants using the app had decreased to 9 (9%), with an average ART adherence rate of 45%. The overall average ART adherence measured by the app after the study was 70%.

When participants in the Mobile group were surveyed about their satisfaction, difficulties, and preferences regarding the app, the results showed that 65% were satisfied or very satisfied with the application, 25% were indifferent, 5% were dissatisfied and 5% did not respond to the evaluation questionnaire. Forty-five per cent (45%) of participants stated that the app significantly or very significantly helped them with ART control. In terms of usability, 59% found the app easy or very easy to use. The most enjoyed features were the medication reminders (65%) and the ability to set personalised reminders for medical appointments (30%). Responses to open-ended questions highlighted the main difficulties encountered while using the app and suggested areas for improvement. More detailed acceptance data can be found in Online Appendix 1.

When non-users were interviewed, 38% cited technical problems with their mobile phones (such as being broken, lost, or having limited memory). Additionally, 22% reported difficulty using mobile phones and apps in general. Twelve per cent (12%) stated that they did not need the app because they already had an established routine for taking ART. Four per cent (4%) expressed fear of being exposed due to their diagnosis, while another 4% either refused the app or showed a lack of interest in it. Seventeen per cent (17%) did not respond.

## Discussion

Although there are several commercially available apps for managing medication intake, most of them have limitations. The free versions of the available apps offer limited resources for medication management. Additionally, many are produced outside Brazil and do not consider Brazilian regulations and patients’ expectations, as most of these apps are presented as a medication control app rather than a health manager. Furthermore, commercially available medication intake reminders do not provide feedback on adherence or personal information from patients and researchers. Similarly, despite message apps such as WhatsApp being widely popular in Brazil, they typically depend on a health agent to manage ART adherence and feedback.

Therefore, to address these issues, our group decided to develop our own ART self-management mobile app adapted for the Portuguese language, and considering Brazilian regulations and specificities. The app prioritises simplicity, featuring an intuitive and unobtrusive interface, requiring minimal storage space on mobile devices, and including automated feedback mechanisms for researchers and patients on adherence to ART. In addition, the app allows users to schedule and manage their medical appointments, while providing real-time updates on certain health parameters, such as BMI, step count, and calorie control. This feature aims to increase acceptability and adherence to the app.

Once the app was developed, we aimed to test its feasibility, acceptability, and effectiveness in managing ART adherence through a single-centre pilot study. Our main findings were that the mobile app did not increase ART adherence or enhance health and clinical outcomes for PLWH. Despite being well received, it was not effective in promoting ongoing use among the participants. Although promising, the use of technology such as mobile apps for ART self-managing remains challenging, especially in a middle-income country such as Brazil. We will address the specific challenges, limitations, and potentials of the app in this discussion.

The lack of effectiveness of a mobile app in enhancing adherence to ART and improving health outcomes for PLWH reflects the findings of other studies. Hightow-Weidman et al.^19^ investigated a gamified mobile app aimed at enhancing engagement to HIV treatment and adherence to antiretrovirals. However, they found no significant effect on improving VL suppression. The authors identified small sample sizes and dropouts as the main limitations of their study. Similar results were described by Himelhoch et al.^20^ In contrast, Perera et al.,^21^ Przybyla et al.,^22^ Horvarth et al.,^23^ and Wood et al.^24^ have shown promising results regarding the use of technology to improve ART adherence.

Perera et al.^21^ successfully showed that an app incorporating personalised health-related visual images and offering real-time information on patients’ medication levels and immunoprotection was an effective intervention for enhancing adherence to ART. The authors did not provide details on socioeconomic and demographic characteristics. Przybyla et al.^22^ found that a smartphone app for daily reporting of substance use and ART adherence among PLWH in the United States (US) was feasible and well received, with high completion rates. However, the authors acknowledged limitations such as the relatively short study period (14 days) and the provision of smartphones to participants.

It is worth noting that while the instruments used to measure ART adherence (CEAT-VIH questionnaire and the Multi-Method Tool) indicated low and medium adherence levels, the majority of participants demonstrated undetectable VL at the beginning of the study. As the two methods used to measure adherence involve self-report questionnaires, the following limitations stand out: the age and level of education of the participants, which may have influenced the results of low and medium adherence observed. In relation to the majority of participants having an undetectable VL, the most recent formulations of ART have significantly improved potency, pharmacokinetic and safety profiles, allowing lower levels of adherence to be sufficient to maintain viral suppression.^4,5,6^ Escobar-Viera et al.^9^ demonstrated that mHealth apps can be effective for improving ART adherence in PLWH with detectable HIV VL and lower CD4+ T-lymphocyte counts. Indeed, initial VL and CD4+ T-lymphocyte counts may be an issue in the effectiveness of a mobile app for improving ART adherence.

Despite being designed with features intended to improve app usage, only 55% of the participants installed the app. Among those who did not install it, the majority cited technical issues with their mobile phones (such as being broken, lost, or having limited memory), or difficulties using mobile phones and apps in general. For those who installed the app but never used it, the main reason reported was difficulty or lack of interest in technology in general. This indicates that access to and incorporation of healthcare technology remains a barrier for many participants. Indeed, it seems that the mHealth model is not accessible to many individuals, not only in middle- to low-income countries but also in high-income countries, given that similar limitations have been described in studies testing mobile technology for ART management in other countries, such as South Africa and the US.^19,25,26^

Close et al.^26^ faced challenges in implementing an app in South Africa, reporting a lack of mobile data, insufficient device space, uninstalling the app, and changing cell phones. Around 37% of the study participants no longer used the cell phone on which the app had been installed during registration. Excluding those who changed phones, uninstalled the app, and deactivated the Global Positioning System, only 53% of the participants had the app working properly during the follow-up. Failures in installing the app were also reported, as some requirements of the US-developed app did not align with the capabilities of many phones used in South Africa. This indicates that implementing the app in real-world conditions and environments with limited resources remains complex. Regarding app abandonment, Hightow-Weidman et al.^19^ reported that in the intervention arm, 40% of participants were using the app at week 13 and this number dropped to 35% at week 26. These results show the difficulty of retaining participants in studies using technology.

It is also important to consider the fatigue resulting from using the app, a common phenomenon in the technology field described by Eysenbach.^27^ The author uses the ‘law of attrition’ to discuss why many mHealth users discontinue use. As with drug testing, mHealth has higher dropout rates, which should be considered a natural and typical characteristic that does not make trials less reliable. Over time, the present study’s participants stopped using the mobile app because of technical difficulties, updating the app, lack of internet connection, and the fact that the participants had been using ART routinely for many years.

Regarding the regular viability and acceptance of the mobile app demonstrated in our study, we hypothesise that age, level of education, and family budget are key characteristics for successfully implementing mobile health. In fact, the participants were of average age (Mobile group: 47 ± 13 vs Control group: 50 ± 13 years), most had more than 8 years of education (Mobile group: 65% vs Control group: 52%), and the majority had a household income of more than one minimum wage (Mobile group: 70% vs Control group: 55%). Therefore, the mobile app used in the present study may be more acceptable among younger and more educated PLWH.

### Limitations

Limitations such as the number of abandonments and reports of PLWH experiencing difficulties with the app were also described by Puig et al.^11^ The authors conducted a 1:1 randomised clinical trial in Spain with 50 elderly HIV patients, in which participants reported poor usability of the app. The study lasted 48 weeks and the patients used the app for an average of 23.7 days. The authors noted that the poor usability and lack of experience with cell phones may explain the infrequent use. In addition, there were no significant changes in quality of life, adherence, or clinical or laboratory parameters among participants that used the app compared to the baseline. Despite this, 91% of participants would recommend using the app and 64.6% considered that the app had improved their healthcare.

The present study presents other limitations that should be addressed. The low number of participants and the lack of randomisation are the main limitations of this study. A higher number of participants would allow us to test acceptability and effectiveness in relation to age, socioeconomic status, and level of education. It is also important to mention the use of self-report questionnaires, and that the majority of participants had an undetectable VL, which suggests good adherence to ART. Therefore, the app has potential for: (1) new and recently diagnosed patients who do not already have a routine for taking their medication; and (2) patients on treatment who do not have good adherence to ART. In contrast, our study also presents some strengths that should be considered. We used a mobile app fully developed by Brazilian researchers, considering the country’s cultural and technological issues, that provides automated feedback for researchers. Unlike studies that considered short periods, we conducted a 90-day test. We also demonstrated that health technology is still poorly funded and used in our country.

## Conclusion

The use of an app – which was available free of charge and operated on Android operating systems – for ART self-management was not effective in improving ART adherence among PLWH and had low viability in this population. However, the app was considered acceptable by the users during this 3-month longitudinal study. We believe that advanced age and low socioeconomic status are critical barriers to testing and using mHealth in Brazil.

## References

[1] Trickey A, May MT, Vehreschild JJ, et al. Survival of HIV-positive patients starting antiretroviral therapy between 1996 and 2013: A collaborative analysis of cohort studies. Lancet HIV. 2017;4(8):349–356. 10.1016/S2352-3018(17)30066-8 PMC5555438 28501495

[2] Trickey A, Sabin CA, Burkholder G, et al. Life expectancy after 2015 of adults with HIV on long-term antiretroviral therapy in Europe and North America: A collaborative analysis of cohort studies. Lancet HIV. 2023;10(5):295–307. 10.1016/S2352-3018(23)00028-0 PMC10288029 36958365

[3] Bekker LG, Smith P, Ntusi NAB. HIV is sexually untransmittable when viral load is undetectable. Lancet. 2023;402(10400):428–429. 10.1016/S0140-6736(23)01519-2 37490934

[4] Byrd KK, Hou JG, Hazen R, et al. Antiretroviral adherence level necessary for HIV viral suppression using real-world data. J Acquir Immune Defic Syndr. 2019;82(3):245–251. 10.1097/QAI.0000000000002142 31343455 PMC6854523

[5] Bezabhe WM, Chalmers L, Bereznicki LR, Peterson GM. Adherence to antiretroviral therapy and virologic failure. Medicine (Baltimore). 2016;95(15):e3361. 10.1097/MD.0000000000003361 27082595 PMC4839839

[6] Stover S, Milloy M, Grant C, et al. Estimating the minimum antiretroviral adherence required for plasma HIV-1 RNA viral load suppression among people living with HIV who use unregulated drugs. AIDS. 2022;36(9):1233–1243. 10.1097/QAD.0000000000003234 35833680 PMC9342903

[7] World Health Organization. mHealth: New horizons for health through mobile technologies. Based on the findings of the second global survey on eHealth. Global Observatory for eHealth series– Volume 3 [homepage on the Internet]. 2011 [cited Dec 2023]. Available from: https://iris.who.int/bitstream/handle/10665/44607/9789241564250_eng.pdf?sequence

[8] Buford E. 30 amazing mobile health technology statistics for today’s physician [homepage on the Internet]. ReferralMD; 2017 [cited Dec 2023]. Available from: https://getreferralmd.com/2015/08/mobile-healthcare-technology-statistics/

[9] Escobar-Viera C, Zhou Z, Morano JP, Lucero R. The Florida mobile health adherence project for people living with HIV (FL-mAPP): Longitudinal assessment of feasibility, acceptability, and clinical outcomes. JMIR Mhealth Uhealth. 2020;8(1):e14557. 10.2196/14557 31913127 PMC6996722

[10] Schaab BL, Remor E. Development, feasibility testing and perceived benefits of a new app to help with adherence to antiretroviral therapy in people living with HIV in Brazil. Pilot Feasibility Stud. 2023;9(1):130. 10.1186/s40814-023-01370-7 37496084 PMC10369752

[11] Puig J, Echeverría P, Lluch T, et al. A Specific mobile health application for older HIV-infected patients: Usability and patient’s satisfaction. Telemed e-Health. 2021;27(4):432–440. 10.1089/tmj.2020.0098 32667858

[12] Orrell C, Cohen K, Mauff K, Bangsberg DR, Maartens G, Wood R. A randomized controlled trial of real-time electronic adherence monitoring with text message dosing reminders in people starting first-line antiretroviral therapy. J Acquir Immune Defic Syndr. 2015;70(5):495–502. 10.1097/QAI.0000000000000770 26218411

[13] Abdulrahman SA, Rampal L, Ibrahim F, et al. Mobile phone reminders and peer counseling improve adherence and treatment outcomes of patients on ART in Malaysia : A randomized clinical trial. PLoS One. 2017;12(5):e0177698. 10.1371/journal.pone.0177698 28520768 PMC5433794

[14] Instituto Brasileiro de Geografia e Estatística. PNAD Contínua–Pesquisa Nacional por Amostra de Domicílios Contínua. 2022;(2):1–12. Available at: https://www.ibge.gov.br/estatisticas/sociais/populacao/17270-pnad-continua

[15] Steel G, Joshi MP. Development of a multi-method tool to measure ART adherence in resource-constrained settings: The South Africa Experience. Arlington, VA: US Agency for International Development; 2007.

[16] Remor E. Valoración de la adhesión al tratamiento antirretroviral en pacientes VIH +. Psicothema. 2002;14:262–267.

[17] Remor E, Milner-Moskovics J, Preussler G. Adaptação brasileira do ‘Cuestionario para la Evaluación de la Adhesión al Tratamiento Antiretroviral’. Rev Saude Publica. 2007;41(5):685–694. 10.1590/S0034-89102006005000043 17713708

[18] Braun V, Clarke V. Using thematic analysis in psychology. Qual Res Psychol. 2006;3(2):77–101. 10.1191/1478088706qp063oa

[19] Hightow-Weidman L, Muessig KE, Egger JR, Vecchio A, Platt A. Epic allies: A gamified mobile app to improve engagement in HIV care and antiretroviral adherence among young men who have sex with men. AIDS Behav. 2021;25(8):2599–2617. 10.1007/s10461-021-03222-y 33740213 PMC9590379

[20] Himelhoch S, Kreyenbuhl J, Palmer-Bacon J, et al. Pilot feasibility study of Heart2HAART: A smartphone application to assist with adherence among substance users living with HIV adherence among substance users living with HIV. AIDS Care. 2017;29(7):898–904. 10.1080/09540121.2016.1259454 28043176 PMC13021365

[21] Perera AI, Thomas MG, Moore JO. Effect of a smartphone application incorporating personalized health-related imagery on adherence to antiretroviral therapy. AIDS Patient Care STDS. 2014;28(11):579–586. 10.1089/apc.2014.0156 25290556 PMC4216527

[22] Przybyla SM, Eliseo-Arras RK, Krawiec G, et al. Feasibility and acceptability of a smartphone app for daily reports of substance use and antiretroviral therapy adherence among HIV-infected adults. AIDS Res Treat. 2016;2016:9510172. 10.1155/2016/95101722016 27610243 PMC5004007

[23] Horvath KJ, Lammert S, MacLehose RF, et al. A pilot study of a mobile app to support HIV antiretroviral therapy adherence among men who have sex with men who use stimulants. AIDS Behav. 2019;23(11):3184–3198. 10.1007/s10461-019-02597-3 31309348 PMC6803067

[24] Wood OR, Schnall R, Kay ES, Jia H, et al. A community health worker and mobile health app intervention to improve adherence to HIV medication among persons with HIV: The CHAMPS study protocol. BMC Public Health. 2023;23(1):1–11. 10.1186/s12889-023-15616-9 37226141 PMC10206576

[25] O’Halloran Leach E, Lu H, Caballero J, et al. Defining the optimal cut-point of self-reported ART adherence to achieve viral suppression in the era of contemporary HIV therapy: A cross-sectional study. AIDS Res Ther. 2021;18(1):36. 10.1186/s12981-021-00358-8 34174904 PMC8234726

[26] Clouse K, Noholoza S, Madwayi S, et al. The implementation of a GPS-based location-tracking smartphone app in South Africa to improve engagement in HIV Care: Randomized controlled trial. JMIR mHealth uHealth. 2023;e44945:11. 10.2196/44945 PMC10238954 37204838

[27] Eysenbach G. The law of attrition. J Med Internet Res. 2005;7(1):1–9. 10.2196/jmir.7.1.e11 PMC1550631 15829473

